# Glucagon-like Peptide-1 Improves Fatty Liver and Enhances Thermogenesis in Brown Adipose Tissue via Inhibiting BMP4-Related Signaling Pathway in High-Fat-Diet-Induced Obese Mice

**DOI:** 10.1155/2021/6620289

**Published:** 2021-04-26

**Authors:** Xingchun Wang, Bingwei Ma, Jiaqi Chen, Hui You, Chunjun Sheng, Peng Yang, Shen Qu

**Affiliations:** ^1^Thyriod Research Center of Shanghai, Shanghai Tenth People's Hospital of Tongji University, School of Medicine, Tongji University, Shanghai 200072, China; ^2^Department of Endocrinology and Metabolism, Shanghai Tenth People's Hospital of Tongji University, School of Medicine, Tongji University, Shanghai 200072, China; ^3^Department of Gastrointestinal Surgery, Shanghai Tenth People's Hospital of Tongji University, School of Medicine, Tongji University, Shanghai 200072, China; ^4^Suzhou Municipal Hospital, Suzhou 215000, Jiangsu, China

## Abstract

**Objective:**

Glucagon-like peptide-1 (GLP-1) receptor agonist is effective in decreasing blood glucose and body weight. It could improve fatty liver with unclear mechanisms. Hence, we aimed to explore whether GLP-1 could improve fatty liver by regulating the BMP4-related signaling pathway.

**Methods:**

Fifteen C57BL/6 mice were randomly assigned to 3 groups. Group A and Group B were fed with a high-fat diet (HFD) to induce fatty liver while Group C was fed with a regular diet (RD) for 24 weeks. Group A and Group B received a subcutaneous injection of exenatide and vehicle (0.9% NaCl), respectively, once daily at doses of 10 nmol/kg during the last 8 weeks. Bodyweight, liver weight, and lipid levels were measured. Histological analyses of liver tissue were performed. The expression of protein and gene measured by western blotting and real-time polymerase chain reaction (RT-PCR) was compared.

**Results:**

Eight-week exenatide treatment significantly decreased body weight in Group A (from 44.08 ± 2.89 g to 39.22 ± 1.88 g, *P* = 0.045). Group A had lower body weight and liver weight than Group B at 24 weeks (39.22 ± 1.88 g vs. 47.34 ± 2.43 g, *P* = 0.001 and 1.70 ± 0.20 g vs. 2.48 ± 0.19 g, *P* = 0.001, respectively). Moreover, Group A showed significantly less liver steatosis than Group B. Additionally, Group A led to slightly decreased serum triglyceride (TG) and cholesterol (TC) levels compared to Group B. Western blotting showed that exenatide could prevent HFD-induced upregulation of BMP4 levels and downstream activation of Smad1/5/8 and the P38 MAPK signaling pathway in the liver. Furthermore, exenatide treatment could reduce BMP4 and enhance UCP-1 (an important thermogenin) in brown adipose tissue (BAT).

**Conclusion:**

Exenatide could improve HFD-induced hepatic steatosis and enhance thermogenesis in BAT, which may be partly attributed to the inhibition of the BMP4-related signaling pathway.

## 1. Introduction

With the change of lifestyle and environment, the incidence of nonalcoholic fatty liver disease (NAFLD) is increasing. Obesity and type 2 diabetes are predisposed to NAFLD [1]. The methods for treatment of NAFLD are limited, mainly through lifestyle and drug intervention to improve insulin resistance and decrease body weight [1]. However, lifestyle change and sustained weight loss are difficult to sustain, and insulin sensitizers have a limited effect on fatty liver disease [2–4]. Therefore, new and effective therapeutic drugs are urgently needed for NAFLD.

Glucagon-like peptide-1(GLP-1) receptor agonists have been proven effective in glycemic control and decreasing body weight [5]. The activation of GLP-1R by exendin leads to suppressed appetite and reduced weight by energy expenditure [6]. GLP-1 treatment can improve NAFLD by decreasing lipid storage and liver enzymes [7, 8]. It may improve the NAFLD and not only indirectly but also directly affect hepatocytes and liver inflammation [9]. However, the mechanisms of GLP-1 receptor agonists on NAFLD have not been explained fully.

Bone morphogenetic protein 4 (BMP4) may be an important factor in obesity and fatty liver. BMP4 plays a key role in regulating adipogenic precursor cell commitment and differentiation [10]. Also, the BMP4 inhibitor can inhibit the differentiation of preadipocytes into mature adipocytes [11]. Adipose tissue includes white adipose tissue (WAT), which stores energy, and brown adipose tissue (BAT), with expenditure of energy. BMP4 may be involved in the pathogenesis of obesity. A recently published article showed that BMP4 promotes differentiation of brown preadipocytes into white-like adipocytes and blunts the activity of mature brown adipocytes [12]. Also, obese T2DM subjects have higher levels of serum BMP4 [12]. BMP4 also regulates glucose metabolism and insulin resistance [13]. Serum BMP4 levels in insulin-resistant mice were elevated [14].

GLP-1 receptor agonists may improve NAFLD. However, the mechanism of GLP-1 receptor agonists on NAFLD is no fully clarified. Therefore, we aimed to explore whether GLP-1 receptor agonists affected the hepatic BMP4-related signaling pathway, thereby improving the fatty liver of obese mice induced by a high-fat diet.

## 2. Materials and Methods

### 2.1. Experimental Design

15 male C57BL/6 mice of 6 weeks weighting 19.08 ± 1.14 g purchased from Shanghai SLAC Laboratory Animal Co. Ltd. (Shanghai, China) were housed in standard cages and maintained in a controlled room condition (Animal House, Shanghai Tenth People's Hospital of Tongji University). The conditions were controlled at about 24°C room temperature, 40 ± 5% relative humidity, and a 12 h:12 h light: dark cycle with dawn/dusk effect. The mice were free to access food and water. The protocol was approved by the Animal Care and Use Committee of Tongji University.

Mice were divided into 3 groups. Group A and B were fed with a high-fat diet (HFD: 5.24 kcal/g, 20% carbohydrates, and 60% fat) for 16 weeks to induce obesity [15]. Group C was fed with a standard diet (STD: 2.9 kcal/g, 6% fat, and 20% protein). After 16 weeks, Group A received exenatide subcutaneous (s.c) injection once daily at a dose of 10 nmol/kg. Group B received one subcutaneous (s.c) injection of 0.9% NaCl of the same dose. The treatment period was 8 weeks. HFD and STD were purchased from Meidisen Biomedical Company (Jiangsu, China). Exendin-4 was purchased from Sigma Aldrich Chemical Co. (St. Louis, MO, USA), dissolved in water. Body weight was measured at the same time every week between 7 p.m. and 8 p.m., at the beginning and after 24 weeks. Liver weight was measured after sacrifice.

### 2.2. Sample Collection

Mice were killed after 24 weeks by decapitation 2 hours after the last injection. Blood samples were collected in tubes with heparin for anticoagulant and centrifuged (10 min at 4°C). The serum was adopted and frozen at −80°C for measurement. Liver and adipose tissue were dissected out and weights were tested after sacrifice. Subcutaneous (inguinal) and visceral (epididymis) white adipose were collected in each group. Brown adipose tissue in the area of the shoulder blade was collected and weighed. Then, the tissue was frozen at −80°C until m RNA and protein distilled for analysis.

### 2.3. Lipid Metabolism and Histological Analyses

The metabolites of serum triglycerides and total cholesterol levels were measured. They were measured spectrophotometrically according to the instructions (NJJCBIO, Jiangsu, China). Paraffin sections of the liver (4*μ* m thick) were processed for hematoxylin-eosin (HE) staining.

### 2.4. Real-Time Reverse-Transcriptase Polymerase Chain Reaction (qRT-PCR)

Total mRNA from the mouse liver was isolated using the Trizol reagent from Invitrogen (Carlsbad, CA). Two micrograms of total RNA were used for cDNA synthesis using an RNeasy kit (QIAGEN) according to the manufacturer's instructions. Quantitative real-time SYBR Green quantitative RT-PCR was performed using a 7900H T real-time PCR system (ABI, CA, USA) to determine the expression of target genes according to the instructions of the SYBR Premix EX Taq (TaKaRa Biotechnology, China). Data were normalized to glyceraldehyde 3-phosphate dehydrogenase (GAPDH) mRNA as endogenous control and analyzed using the ΔΔCt method. Using the ΔΔCt method, the ratio of the mRNA expression level of the target gene to endogenous control GAPDH was 2-△C (*T*), where △C (*T*) = C (T)target gene –C (*T*) GAPDH. Primer sequences are shown in Table 1.

### 2.5. Western Blotting

Expression of total Smad and phospho-Smad (p-Smad), total P38 and phosphor-P38 (p-P38) and BMP4, and UCP-1 was measured by Western blotting. Protein was separated on 10% SDS-PAGE gels and transferred onto nitrocellulose membranes. Antibodies of BMP4, Smad, p-Smad, P38, and p-P38 were purchased from Cell Signaling Technology (USA). The antibody of UCP-1 was purchased from ABclonal (USA). Antibodies of *α*-tubulin and GAPDH were purchased from Proteintech (USA). BMP4, P38, p-P38, p-Smad, Smad, and UCP-1 expression were analyzed using a rabbit-antibody (1 : 1000) primary antibody, and a rabbit-anti mouse IgG-HRP (1 : 1000) secondary antibody. GAPDH and *α*-tubulin (1 : 1000 dilution for the primary antibody and 1 : 1000 dilution for the secondary antibody) were used as controls. Western blot bands were scanned and quantified by an image analyzer, Quantity One System (Odyssey, USA).

### 2.6. Statistical Analysis

Statistical analysis was performed using SPSS software version 20.0 and GraphPad Prism software (GraphPad Software Inc). Continuous values were expressed as mean ± standard deviation, and the counting data were expressed as number(n). *t*-test was used for data of normal distribution, and a nonparametric test was used for data that was not a normal distribution. Independent samples *t*-test was used for comparison of independent samples and one-way ANOVA was used for comparison of multiple data. A paired sample *t*-test was used for before-and-after comparison. A value of *P* < 0.05 was considered statistically significant.

## 3. Results

### 3.1. Body Weight of Three Groups

**Figure 1:**
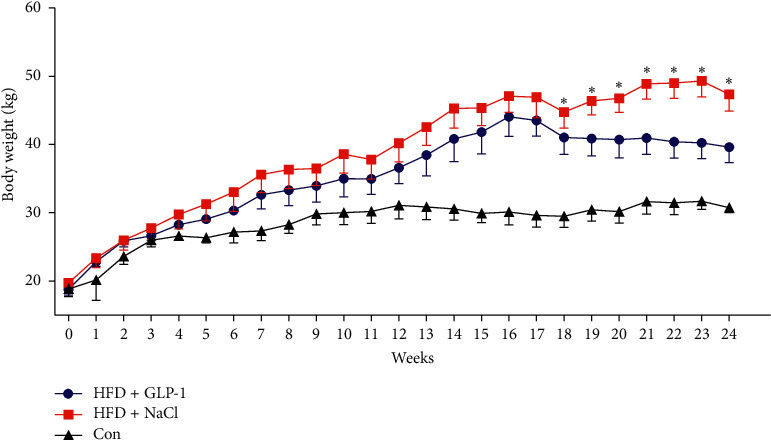
Comparison of body weight in each group. Group A: HFD + GLP-1; Group B: HFD + 0.9% NaCl; Group C: Con. ^∗^*P* < 0.05 vs. Group A.

As shown in Figure 1, the body weights of HFD mice were significantly increased compared to RD at 16 weeks (Group A vs. Group C: 44.08 ± 2.89 g vs. 30.12 ± 1.89 g; Group B vs. Group C: 47.08 ± 2.35 g vs. 30.12 ± 1.89 g, all *P* < 0.001). The average body weight of HFD mice was about 42.3% over the RD group. There were no significant differences between the two HFD groups at 16 weeks (44.08 ± 2.89 g vs. 47.08 ± 2.35 g, *P* = 0.111). However, after 8 weeks of intervention, the body weight of HFD mice was significantly decreased in the GLP-1 treatment group (from 44.08 ± 2.89 g to 39.62 ± 2.26 g, *P* = 0.043). There was no significant change of body weight in the compared group treated with NaCl (from 47.08 ± 2.35 g to 47.34 ± 2.43 g, *P* = 0.897). Body weight was significantly lower in the GLP-1 treatment group than the compared group at 24 weeks (39.62 ± 2.26 g vs. 47.34 ± 2.43 g, *P* = 0.001).

### 3.2. Liver of Three Groups

**Figure 2:**
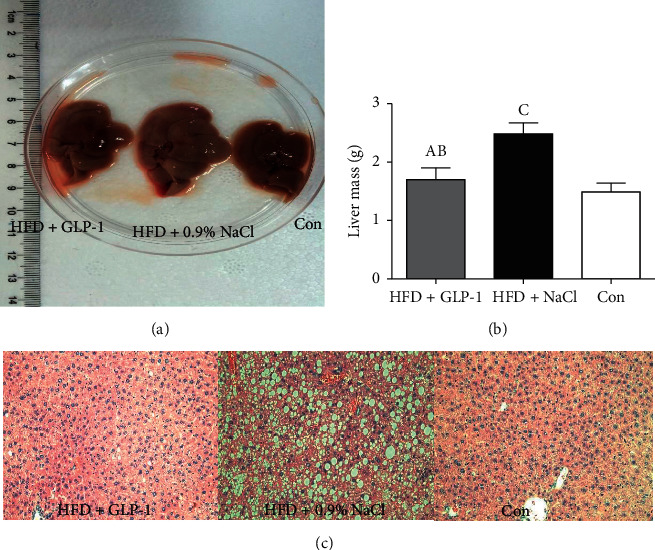
Comparison of livers among groups. Group A: HFD+GLP-1; Group B: HFD+NaCl; Group C: Con. (a) *P* = 0.001 vs. Group B; (b) *P* = 0.161 vs. Group C; (c) *P* < 0.001 vs. Group C.

Livers were removed and liver mass was measured upon sacrificed. As shown in Figure 2, lipid deposition in the liver was observed in the HFD group in both gross specimens and HE staining sections. Compared with the HFD group with NaCl intervention, the GLP-1 intervention group showed significant improvement in fatty liver and lipid droplet deposition. The weight of the liver was also significantly lighter in the GLP-1 intervention group than the control group (1.70 ± 0.20 g vs. 2.48 ± 0.19 g, *P* = 0.001). There was no significant difference in liver weight between the HFD + GLP-1 group and the RD diet group (1.70 ± 0.20 g vs. 1.49 ± 0.15 g, *P* = 0.161).

### 3.3. Comparison of White Adipose Tissue in Mice

**Figure 3:**
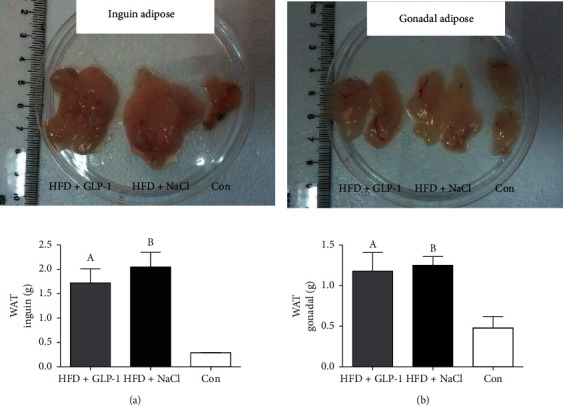
Comparison of white adipose tissues among three groups of mice. Group A: HFD+GLP-1; Group B: HFD+NaCl; Group C: Con. (a) A: *P* = 0.001 vs. Group C; B: *P* = 0.001 vs. Group C. (b) A: *P* = 0.035 vs. Group C; B: *P* = 0.028 vs. Group C. WAT: white adipose tissue.

As shown in Figure 3, mice in the HFD groups had significantly more inguinal adipose than those in the control group (Group A: Group C = 1.72 ± 0.29 g:0.29 ± 0.004 g; Group B: Group C = 2.05 ± 0.30 g:0.29 ± 0.004 g, all *P* < 0.001). The gonadal adipose in the HFD group was also significantly higher than that in the control group (Group A: Group C = 1.18 ± 0.23 g:0.48 ± 0.14 g, *P* = 0.035; Group B: Group C = 1.25 ± 0.11 g: 0.48 ± 0.14 g, *P* = 0.028). Inguinal and gonadal adipose of GLP-1 treated group were slightly less than those in group B without significant difference (*P* = 0.151 and *P* = 0.394).

### 3.4. Lipid Metabolism in Mice

**Figure 4:**
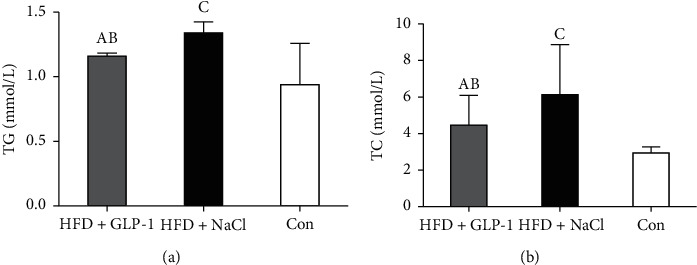
Lipid metabolism of three groups. Group A: HFD+GLP-1; Group B: HFD+NaCl; Group C: Con. (a) A: *P* = 0.022 vs. Group B; B: *P* = 0.265 vs. Group C; C: *P* = 0.047 vs. Group C. (b) A: *P* = 0.286 vs. Group B; B: *P* = 0.077 vs. Group C; C: *P* = 0.036 vs. Group C. TG: triglyceride; TC: total cholesterol.

As shown in Figure 4, serum triglyceride levels in the HFD group treated with GLP-1 were significantly lower than those in the HFD group treated with normal saline (*P* = 0.022). There was no significant difference in serum triglyceride levels between the GLP-1 group (Group A) and the RD group (Group C) (*P* = 0.265), while the serum triglyceride levels in Group B were significantly higher than those in Group C (*P* = 0.047). Serum cholesterol levels in Group B were significantly higher than those in Group C (*P* = 0.036), while the serum cholesterol levels in Group A were not significantly different from Group C (*P* = 0.077) and were slightly lower than those of Group B (*P* = 0.286).

### 3.5. BMP4 mRNA Expression in the Liver

**Figure 5:**
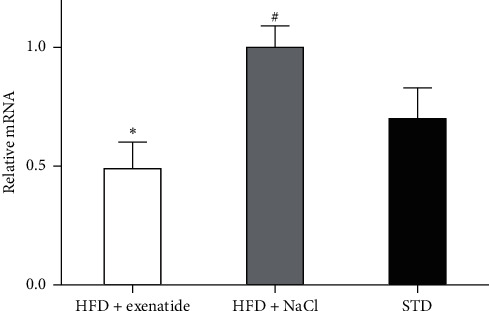
Comparison of BMP4 mRNA expression in the livers of three groups. BMP4: bone morphogenetic protein 4. ^∗^*P* < 0.05 for Group A vs. Group B; ^#^*P* < 0.05 for Group B vs. Group C.

As shown in Figure 5, the expression of liver BMP4 mRNA levels in the GLP-1 treatment group was significantly lower than that in the control group with normal saline intervention.

### 3.6. Protein Expression of BMP4 and BMP4-Related Signaling Pathway

**Figure 6:**
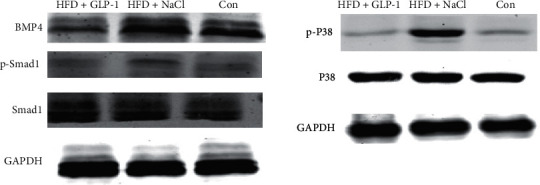
Protein expression of BMP4 and related signaling pathway in liver. Group A: HFD+GLP-1; Group B: HFD+NaCl; Group C: Con. BMP4: bone morphogenetic protein 4.

As shown in Figure 6, the total Smad1/5/8 and P38 in the three groups showed no significant difference, while the phosphorylated Smad1/5/8 and P38 in the HFD + GLP-1 group (Group A) were significantly lower than those in the HFD + NaCl group (Group B), and the expression of BMP4 protein was also significantly lower in Group A than Group B. This indicates that GLP-1 may reduce the expression of BMP4 in the liver and inhibit BMP4-related signaling pathways.

### 3.7. Thermogenesis and BMP4 Expression in Brown Adipose Tissue

**Figure 7:**
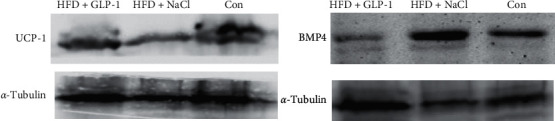
Expression of BMP4 and thermogenic protein in brown adipose tissue. Group A: HFD+GLP-1; Group B: HFD+NaCl; Group C:Con. BMP4: bone morphogenetic protein 4; UCP1: uncoupling protein-1.

As shown in Figure 7, HFD mice had decreased heat production, and the expression of uncoupling protein-1 (UCP-1) in brown adipose tissue was significantly decreased, while GLP-1 treatment increased UCP-1 expression in brown adipose tissue. In addition, GLP-1 treatment significantly reduced the expression of BMP4 in brown adipose tissue.

## 4. Discussion

NAFLD is caused by excessive fatty degeneration in liver cells except for the consumption of alcohol, viral infection, autoimmunity, and drugs. NAFLD is an independent risk factor for cardiovascular risk [16, 17]. NAFLD is associated with insulin resistance and dyslipidemia, which further exacerbates fat deposition in the liver [18, 19]. Lifestyle can reduce body weight and improve insulin sensitivity of the patients with NAFLD [20–22]. The main therapeutic drugs for NAFLD are insulin sensitizers, such as metformin and thiazolidinediones [4, 23]. With the limited effects of lifestyle invervention and insulin sensitizers,effective methods are needed to treat NAFLD.

GLP-1 is a 30-amino acid peptide secreted by Langerhans cells of the jejunum terminal, ileum, and colonic epithelium, which can promote the release of insulin, increase the sensitivity of insulin, delay gastric emptying, and suppress appetite [24]. The synthesized GLP-1 receptor agonist resists the action of dipeptidyl peptidase-IV and prolongs the half-life of GLP-1 [24]. GLP-1 receptor agonists have been verified efficacious in decreasing blood glucose by stimulating glucose-dependent insulin secretion, reducing glucagon release as well as improving metabolism. It is beneficial for controlling body weight and dyslipidemia and improving fat distribution and hepatic steatosis. GLP-1 receptor agonists may improve NAFLD indirectly by reducing body weight, increasing insulin sensitivity, or directly binding GLP-1 receptor on liver cells. Human liver cells have GLP-1 receptors [25]. GLP-1 binds to GLP-1 receptor in hepatocytes and plays a direct role in lipid metabolism. The indirect therapeutic effect of GLP-1 receptor agonists on NAFLD is achieved by reducing body weight and increasing insulin sensitivity. In addition, studies have shown that 4 weeks of exendin-4 therapy can significantly reduce hepatic triglyceride content without changing body weight [26], and exendin-4 can inhibit the accumulation of triglyceride in primary hepatocytes induced by palmitic acid, thus exerting a direct effect on the improvement of NAFLD [27]. The mechanism of GLP-1 receptor agonists in the treatment of fatty liver has not been yet fully clarified. In our study, GLP-1 receptor agonists led to the improvement of NAFLD in the mice fed with HFD when compared to the control group.

Bone morphogenetic proteins (BMPs) are numbers of the transforming growth factor-beta (TGF-*β*) family [10, 28]. It plays a key role in regulating biological function during embryonic development and adult tissue homeostasis such as intestine, muscle, kidney, and brain, as well as adipose tissue [29, 30]. BMP4 binds to type II and type I serine-threonine kinase receptors and then signal to transduce through Smad and Smad-independent signaling pathways [31]. The Smad signaling pathway is transduced by the transcription factors Smad1/5/8 and the Smad-independent signaling pathway is mediated by, for example, p38 mitogen-activated protein kinase (MAPK) and JNK [32]. The BMP4 signaling pathway is involved in the pathophysiology of obesity and abnormal glucose metabolism [33]. In tissues and organs targeted by insulin, such as fat, liver, and skeletal muscle, insulin and BMP4 signaling pathways directly interacted to coordinate energy balance. BMP4 inhibits the uptake of glucose in the petri dish by liver cells [13]. BMP4 significantly increased the expression of serine phosphorylation expression of insulin receptor substrate-1 (IRS-1) and directly inhibited the insulin signaling pathway of the liver, adipose tissue, and skeletal muscle by activating PKC-*θ*, leading to insulin resistance [13]. BMP4 pathway plays a role in the generation of fat, participates in the regulation of fat metabolism and insulin resistance, and affects the insulin signaling pathway of the liver. This study investigated whether GLP-1 receptor agonist improves fatty liver by influencing the expression of BMP4 in the liver and regulating BMP4-related signaling pathways. The results showed that there was no significant difference in total Smad1 and P38 expression among groups, while the phosphorylation of Smad1 and P38 in the GLP-1 treated group was significantly decreased compared to the HFD control group, and the expression of BMP4 protein was also significantly decreased. Therefore, it was speculated that GLP-1 might improve the fatty liver of mice by reducing the expression of BMP4 protein in the liver and downregulating Smad1 and P38 signaling pathways.

BAT thermogenesis is a conserved mechanism to maintain body temperature, and BAT contribution to energy expenditure can represent a relevant modulator of metabolic homeostasis. BMP4 plays a role in the production of brown adipose tissue. Overexpression of BMP4 in brown adipose tissue in mice induces the transformation of brown adipose tissue into white adipose tissue and reduces the thermogenesis in mature brown adipose [12]. In this study, we explored the expression of UCP-1 and BMP4 in brown adipose tissue. The result showed that the expression of UCP-1 in brown adipose tissue in mice fed with high fat was significantly decreased, indicating that the thermogenesis in brown was descended with a high-fat diet. Additionally, a previous study showed that GLP-1R agonist stimulates BAT thermogenesis and adipocyte browning independent of nutrient intake in mice [34]. The central mechanism may involve the activation of AMPK located in the hypothalamic ventromedial nucleus (VMH) [34]. It may reduce local adipogenesis, improve fat utilization, and induce brown fat differentiation [35]. Our study showed that GLP-1 treatment significantly increased the expression of UCP-1 in brown adipose tissue. Meanwhile, the expression of BMP4 was reduced in brown adipose tissue in the GLP-1 treated group when compared to the control group. These results indicate that GLP-1 may increase thermogenesis by decreasing the expression of BMP4.

In conclusion, GLP-1 receptor agonists could decrease body weight and liver fat deposition in HFD mice. The effect of GLP-1 receptor agonists on the fatty liver may be achieved by reducing the expression of BMP4 and downregulating the Smad and P38 signaling pathway. Additionally, GLP-1 receptor agonist enhanced thermogenesis in brown adipose tissue, which may be partly attributed to the inhibition of BMP4 expression.

## Figures and Tables

**Table 1:** Primer sequence of real-time PCR.

Primer name	Sequence (5′–3′)	Length
F-MUS-BMP4	TCTCCGTCCCTGATGGGATT	20
R-MUS-BMP4	TGGTGTCTCATTGGTTCCTGC	21
F-MUS-GAPDH	AGGTCGGTGTGAACGGATTTG	21
R-MUS-GAPDH	TGTAGACCATGTAGTTGAGGTCA	23

## Data Availability

The datasets used and/or analyzed during the current study are available from the corresponding author on reasonable request.
